# Resistance to obesity prevents obesity development without increasing spontaneous physical activity and not directly related to greater metabolic and oxidative capacity

**DOI:** 10.1371/journal.pone.0271592

**Published:** 2022-08-11

**Authors:** Jóctan Pimentel Cordeiro, Daniel Sesana da Silva, Suellem Torezani-Sales, Amanda Rangel Madureira, Erick Roberto Gonçalves Claudio, Danilo Sales Bocalini, Ana Paula Lima-Leopoldo, André Soares Leopoldo

**Affiliations:** 1 Center for Physical Education and Sports, Experimental Physiology and Biochemistry Laboratory, Federal University of Espírito Santo, Vitória, Espírito Santo, Brazil; 2 Postgraduate Program in Nutrition and Health, Health Sciences Center, Federal University of Espírito Santo, Vitória, Espírito Santo, Brazil; 3 Postgraduate Program in Physiological Science, Federal University of Espírito Santo, Vitória, Espírito Santo, Brazil; Case Western Reserve University School of Medicine, UNITED STATES

## Abstract

There are evidence that obese-resistant animals are more physically active, due to a higher rate of lipid oxidation. Efficiency in such pathways can favor greater spontaneous physical activity and, consequently, less body fat deposition. The aim of study was characterizing the nutritional profile and spontaneous physical activity in the condition of Resistance to Obesity (OR). *Wistar* rats were randomized into standard diet (SD; n = 50) and high-fat diet (HFD; n = 50) groups, after obesity induction, were redistributed into Control (C), False-control (FC), Propensity to obesity (OP) and OR, and then spontaneous physical activity was evaluated. Analyzed parameters: body mass (BM), epididymal (EF), retroperitoneal (RF), visceral (VF) and respective summations (∑), adiposity index (AI), nutritional, morphological, biochemical and metabolic parameters and protein quantification. The comparison of the groups was performed by ANOVA one or two factors, with 5% significance adopted. OP and FC presented high final MC values compared to C and OR. OR had lower EF, RF, VF, ∑ and IA compared to OP. OR had similar values to C and higher HDL than FC and OP. In GTT, OR and C presented similar values and both were lower than OP in the 30 minutes. OP promoted higher values than C for glycemic AUC. OR had higher PPARγ content than C and OP, as well as levels similar to C for leptin and insulin. Spontaneous physical activity did not differ between groups. The results were not enough to show that OR animals have greater lipid oxidative capacity, as well as greater spontaneous physical activity.

## Introduction

Resistance to Obesity (OR) is a growing research topic in the scientific community with a considerable increase in the number of publications in recent years [1–3]. However, the etiology of OR is still unknown in the literature, since its characterization was described more than three decades ago. In this sense, most of the works dedicate efforts to the study of metabolism and metabolic pathways related to the accumulation and/or oxidation of adipose tissue alone [2–4] or associated with spontaneous physical activity [5–7], but the study of lipid oxidation pathways in skeletal muscles in models of OR still seem to be scarce.

According to Levin & Sullivan [8], OR is associated with the complex interaction of several metabolic and environmental factors and confers the ability to lower weight gain and deposition of body fat even when ingesting high-calorie diets [4,9]. It is important to note that studies that use the identification of obesity resistance from diets present a closer approximation of the characteristics observed in human beings [10], considering that lean phenotypes are observed in some individuals and not in others, even with similar dietary patterns and level of physical activity [9].

In this context, the lower metabolic capacity of OR animals to convert ingested food into body mass gain is confirmed by the lower final body mass [11,12], as well as keeping the main factors related to accumulation of adipose tissue. Akieda-Asai et al. [4], when analyzing the cross-sectional area (CSA) of white adipose tissue (WAT) adipocytes in OR animals, observed CSA similar to control animals and significantly lower CSA than obese prone (OP) animals, as well as lower expression of fatty acid synthase (FAS) enzyme and increased expression of the translocator protein carnitine palmitoyl transferase (CPT-1), These findings support the theory that OR leads to lower fat deposition due to decreased lipogenesis and increased lipid oxidation, respectively [4]. Therefore, OR rats supposedly have increased lipid oxidation capacity and this characteristic seems to be associated with energy metabolism visualized by greater expression of proteins responsible for the transport of fatty acids (FA) to the mitochondria [4] where they will be metabolized in β-oxidation and in the Krebs cycle [13].

However, it is not clear why energy generation is increased in WAT in the condition of OR, given that adipocytes are not working cells, therefore they do not have high demands for adenosine triphosphates (ATP), a fact that is justified by lower mitochondrial density of this tissue compared to skeletal muscles. Working cells have greater FA oxidative capacity, both due to greater mitochondrial density and greater energy demand [13]. Under high availability of lipids, skeletal muscles predominantly use FA for the synthesis and obtainment of ATP [13], so that when the effort and duration of muscle work increase, the use of FA will be greater, for example, in physical exercise.

Considering these concepts of muscle work and lipid oxidation, mentioned above, some authors tested the hypothesis that OR rats would have lower body fat deposition due to greater spontaneous physical activity (SPA) [6]. Researchers, using devices with 45 infrared activity sensors capable of detecting horizontal, vertical and ambulatory movements, showed that OR animals showed greater vertical activity, which indicates that they are more active than obese ones [7]. These findings suggest that this factor contributes to lower levels of adiposity in OR [6]. However, the study does not demonstrate the metabolic pathways or mechanisms involved, which could elucidate the priority of using FA as an energy substrate and, consequently, favor the characterization of OR in these animals.

Even though there are few studies in the literature about the metabolic processes that explain OR, these studies support the hypothesis that OR animals are more physically active due to their greater capacity to generate ATP from lipid oxidation. Novak, Kotz & Levine [7] corroborate this information by demonstrating that OR rats have a lower respiratory quotient (RQ), which per se is indicative of the efficiency of metabolizing FA as an energy substrate. Therefore, animals that present better efficiency of lipid oxidation pathways can develop greater physical performance.

In this sense, the characterization of morphological and metabolic parameters, as well as the levels of physical activity and the lipid utilization pathways, as energy substrates for ATP generation, apparently predominant in OR animals, may be the key to understanding possible interventions and/or scientific and technological development that enable the creation and improvement of strategies to combat obesity and increase human performance. Therefore, it is necessary to investigate the expression of proteins responsible for the transport of lipids to the mitochondria, as well as proteins that regulate energy generation pathways from fat in the skeletal muscles of OR rats. Furthermore, mapping the hormonal and metabolic profile of rats with inversely proportional characteristics (OR *vs*. FC) may elucidate future intervention strategies against excessive body weight gain.

Thus, the objective was to characterize metabolically and morphologically the condition of OR, as well as to investigate the probable pathways responsible for lipids oxidation. In addition, to investigate whether OR rats show increased spontaneous physical activity related to greater metabolic and oxidative capacity. Therefore, the hypothesis of this study is that OR rats have reduced levels of adiposity due to greater capacity for lipid oxidation as a form of energy production, indicating that these animals develop greater spontaneous physical activity.

## Material and methods

### Animal care

Thirty-day-old *Wistar* rats, obtained from the Animal Quarters of the Federal University of Espírito Santo (Vitória, ES, Brazil), were individually caged in rooms with regulated temperature (24 ± 2°C), humidity (55 ± 5%) and inverted lighting cycle (12h/12h). The experimental procedures were carried out in accordance with the "*Guide for the Care and Use of Laboratory Animals*" published by the "U.S. National Institutes of Health” and approved by the Ethics Committee on Animal Use (CEUA) of the Federal University of Espírito Santo (N^o^. 12–2018).

### Experimental protocol

After a period of seven days for acclimation, the rats were initially randomized into two groups: 1) fed a standard diet (SD, n = 50) and; 2) fed a saturated high-fat diet (HFD, n = 50). The SD animals received a standard rodent diet containing 13.9% of their calories from fat, 55.9% from carbohydrates and 30.2% from protein (Nuvilab CR1-Nuvital). HFD rats received a saturated high-fat diet containing 37.6% of its calories from fat, 44.6% from carbohydrates and 17.8% from proteins (Nutriave Alimentos®, Viana, Espírito Santo, Brazil), as which have the same nutritional composition, with the exception of flavoring additives. These experimental diets provided sufficient amounts of protein, vitamins, and minerals according to the Nutrient Requirements of Laboratory Animals [14].

All animals had free access to water and chow (40 g/day), and daily food consumption was measured. The feed efficiency (FE) was calculated by dividing the total weight gain of the animals (g) by the total ingested energy (kcal) [15,16]. Caloric intake was calculated by daily food consumption multiplied by the caloric value of each diet (g x kcal).

**Fig 1:**
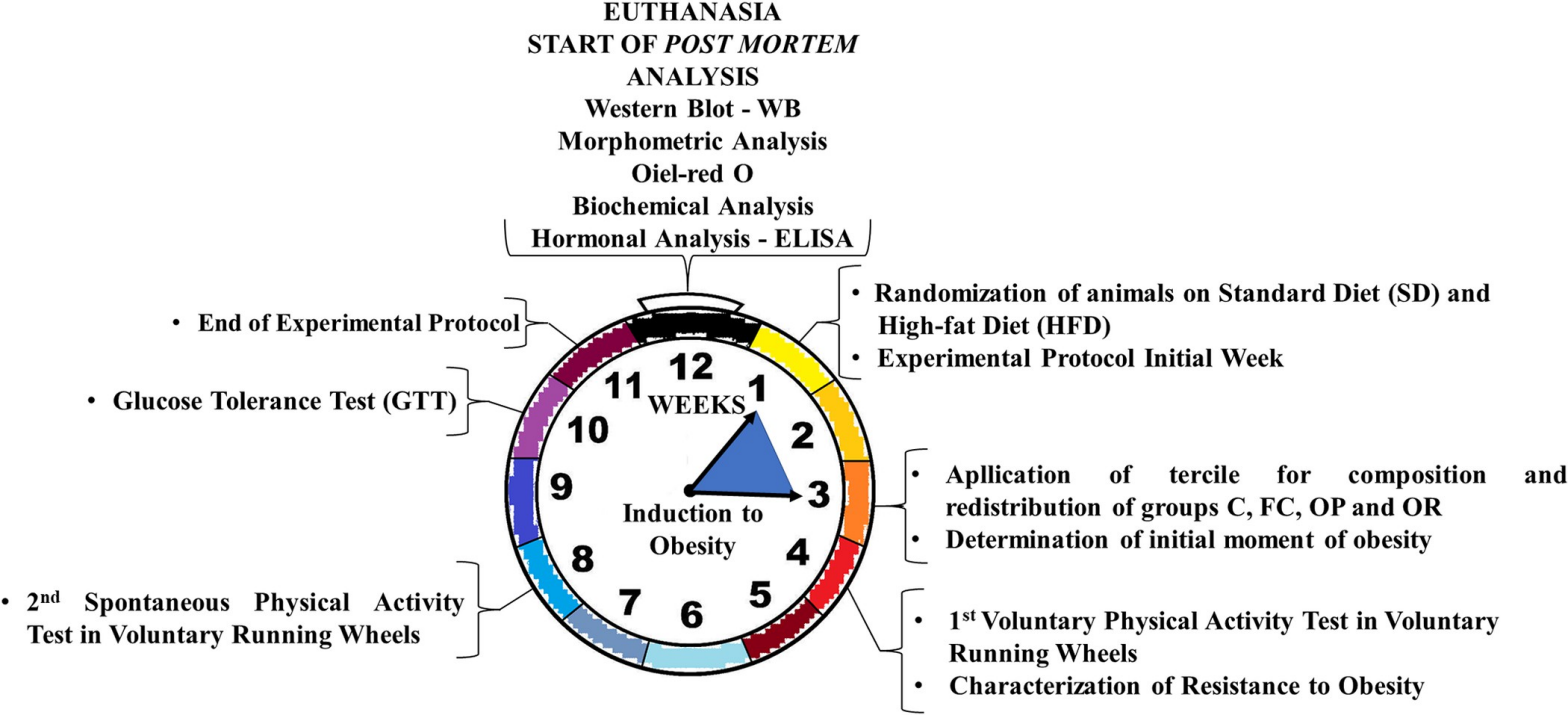
Schematic representation of the experimental protocol (12 weeks; *in vivo* as well as *post mortem* analyses).

The experimental protocol consisted of a total period of 11 weeks, divided into two stages, as shown in Fig 1: induction (3 weeks) and maintenance of obesity and characterization of OR (8 weeks). The initial moment of obesity (3 weeks) was determined as previous studies carried out by our laboratory [17,18], being considered the initial moment of obesity when there was a significant increase in body weight of HFD rats in relation to SD rats.

### Criteria for composition and redistribution of groups

At the initial moment of obesity (3 weeks), in order to form homogeneous groups, one criterion based on body weight was used to redistribute the SD and HFD groups in Control (C), False-Control (FC), Obese Resistant (OR) and Obese Prone (OP). This criterion consisted of organizing the animals in each group in ascending order according to body weight, and the cutoff point was identified in the animals by terciles: 1) lower body weight; 2) intermediate body weight; 3) animals with greater body weight. Thus, animals fed with SD and HF diets, and which presented lower body weight, were classified as Control (C, n = 15) and Obese Resistant (OR, n = 16), respectively. Likewise, the animals that received the respective diets and that presented greater body weight were classified as False-Control (FC, n = 16) and Obese Prone (OP, n = 16). Animals with intermediate body weight were excluded from the study (SD, n = 19; HFD, n = 18).

### Spontaneous physical activity (SPA)

Spontaneous physical activity was analyzed using voluntary running wheels with a spin counter, coupled to the animal’s box, according to a protocol adapted Teske, Billington & Kotz [6].

After redefining the groups in C, FC, OP and OR, in the 4^th^ week of the experimental protocol, 10 rats from each group were randomly selected to perform the test. One animal from each group was placed in the box for 07 uninterrupted hours, with access to water and feed, as well as free access to the wheel. After 07 hours, the number of counts performed was measured [6]. In the next dark cycle, another animal from each group underwent the same procedure until all 10 had performed the test. At 8^th^ week, the same procedure was performed, but for 4 uninterrupted hours.

### Glycemic profile

In the 10^th^ week of treatment, the rats underwent a 6-hour fasting period. Blood samples from the tail artery were collected at baseline and after intraperitoneal administration of 25% glucose (Sigma-Aldrich,®St Louis, MO, USA), equivalent to 2g/kg. Blood samples were collected at time 0, considered baseline condition, and after 30, 60, 90 and 120 minutes of glucose administration. Glucose tolerance in these animals was assessed by the area under the curve for glucose [1].

### Euthanasia

After the end of the experimental protocol (week 12), the animals were fasted for 12–15 hours, and anesthetized with an intraperitoneal injection (ip) containing ketamine hydrochloride (50 mg/kg/intraperitoneal; DOPALEN®, Sespo Indústria e Comércio Ltda., Vetbrands Division, Jacareí, São Paulo, Brazil) and xylazine hydrochloride (10 mg/kg/intraperitoneal; ANASEDAN®, Sespo Indústria e Comércio Ltda., Vetbrands Division, Jacareí, São Paulo, Brazil); when the animal did not have an adequate anesthetic plan for the surgical intervention, a higher single dose was administered (20 to 30% of the initial dose of anesthetics). After euthanasia, the animals were submitted to median thoracotomy. Blood samples were collected in Falcon tubes, centrifuged at 3,000 rpm for 10 minutes (Eppendorf® Centrifuge 5804-R, Hamburg, Germany) and the serum stored in a freezer at -80°C (Thermo Fisher Scientific LLC, Asheville, NC, USA). Epididymal, retroperitoneal and visceral (mesenteric fat) fats deposits, liver and soleus skeletal muscle was dissected. After collection, samples destined for molecular analysis (visceral fat and soleus skeletal muscle) were frozen in liquid nitrogen and stored in a freezer at -80°C. The samples destined for morphological analysis (liver and retroperitoneal and visceral fats) were stored in microtubes containing *Karnovsky* solution. Part of the liver was used to determine tissue water content.

### Nutritional profile assessment

The nutritional profile was determined by analyzing body weight (BW), body fat (BF) and adiposity index (AI). The BW was measured weekly, and the amount of body fat was determined by the sum of epididymal, retroperitoneal and visceral (mesenteric fat) fat deposits. AI was calculated by dividing the total body fat by the final body weight, multiplied by 100, as applied in the study by Cordeiro et al. [1].

### Biochemical and hormonal profile

After euthanasia at week 12, serum concentrations of glucose, triacylglycerol, total cholesterol, alanine aminotransferase (ALT) and aspartate aminotransferase (AST) were determined using specific kits (Bioclin Bioquímica®, Belo Horizonte, Minas Gerais, Brazil and Synermed do Brasil Ltda, São Paulo, Brazil) and analyzed using colorimetric enzymatic tests using an automated biochemical analyzer BS-200 (Mindray do Brasil—Comércio e Distribuição de Equipamentos Médicos Ltda, São Paulo, Brazil). In addition, the same serum has been used to perform the hormonal profile (leptin and adiponectin), using specific kits (# EZRL-83K and # RAB1136, Millipore, Sigma Aldrich, Spruce Street, Saint Louis, MO, USA) and ELISA method with the aid of a microplate reader (Biochrom EZ Read 800 Plus, Holliston, MA, USA). Analysis of serum insulin concentrations, previously collected at the time of euthanasia, were outsourced and performed by the Central Laboratory for High Performance Technologies in Life Sciences (LACTAD-UNICAMP/SP/Brazil) using the Multiplex Immunoassay technique (MILLIPLEX ® MAP Rat Metabolic Magnetic Bcad Pael, #RMHMAG- 84K).

### Morphological analysis

Retroperitoneal and visceral white adipose tissue, as well as liver samples were removed, weighed and stored in tube containing *Karnovsky* solution for 48h. For this purpose, the fragments of adipose tissue were dehydrated in alcohol, clarified in xylol and embedded in paraffin. Histological sections 4 μm thick were made, stained in a hematoxylin-eosin (HE) solution and projected at 40 times magnification with the aid of a microscope (Leica Mikroshopie & System GmbH, Wetzlar, Germany), coupled with a video camera, which sends digital images to a computer equipped with an image analysis program (Image Pro-plus, Media Cybernetics, Silver Spring, Maryland, USA). Images were analyzed with specific software (Image J, v 1.43u, National Institute of Health, USA). To calculate the cross-sectional area (CSA) of adipose tissues, 50 to 70 cells of each sample were measured. Tissues should be cross-sectioned and have a rounded shape with a visible nucleus in the center of the cell. The CSA were used as indicators of cell size, characterizing the presence or absence of adipocyte maturation.

Liver tissue samples were inserted into a container filled with Tissuetek OCT composite gel (Sakura Finetek, CA). The samples were sectioned at a thickness of 10 μm in a cryostat at -25°C (Jung CM 1860; Leica, Wetzlar, Germany). Sections were mounted on gelatin-coated slides and stained with Oil-Red-O (Sigma-Aldrich, St. Louis, MO) for detection of neutral lipids. Images were captured with a video camera (AxioCam ERc5s, Carl Zeiss, Germany) coupled to an optical microscope (AX70, Olympus Corporation, Japan) using 40 times objective and quantified using Image J software (National Institutes of Health, Bethesda, USA). For each analysis, 10 different fields per animal were used randomly to calculate the average percentage of stained area.

### Determination of water content in hepatic tissue

The evaluation of tissue water content was performed in samples of liver. After removing the tissue to be examined, the fresh weight was measured. Afterwards, the samples were submitted to drying in an oven at a temperature of 55 ± 5°C, for a period of 48 hours. The determination of the water content was expressed in relative values and calculated by the following formula: [(*FW*-*DW*)/*FW*] x 100%, where FW represents the fresh weight and the dry weight (DW).

### Western blot analysis

The homogenization of frozen soleus muscle was performed in tubes containing lysis buffer (10mM Tris-HCL at pH 7.4, 1mM NaVO3, 1% SDS, 0.05mM DTT, 5mM EDTA, 1mM PMSF, 10mM NaF) added with an inhibitor of protease (1:100), in the proportion 1mL/100mg. The samples were centrifuged for 20 minutes at 14,000 rpm at 4°C (). The pellet formed was discarded and the protein concentration of the supernatant quantified by the Bradford method [19]. The absorbance was measured at 595 nm using a spectrophotometer. Then, aliquots were prepared containing the necessary volume for a 50 μg protein load, in addition to the sample buffer (Laemmli 4×). The samples were loaded onto 10% SDS-polyacrylamide gels, and then electrophoresis was performed. Subsequently, the protein was transferred electrophoretically to a polyvinylidene fluoride membrane (Bio-Rad, CA, USA); the membranes were incubated for 1 hour, at room temperature, with 5% bovine serum albumin blocking solution and were incubated overnight at 4°C, under agitation, with the primary antibodies.

For skeletal muscle, monoclonal antibody anti-mouse PPARγ (1:500, Santa Cruz Biotechnology) and polyclonal antibody anti-rabbit UCP-3 (1:1000, ABCAM) were used. For adipose tissue, the polyclonal antibody anti-rabbit protein HSL (1μg/mL, ABCAM) was used. The normalizing antibody used for both tissues was the anti-mouse β-actin monoclonal antibody (1:1000, Cell Signaling). The binding of the primary antibody was detected with secondary antibodies conjugated with peroxidase anti-mouse IgG (1:10000, Santa Cruz Biotechnology) or an anti-rabbit IgG (1: 10000, Santa Cruz Biotechnology), and the detection of the bands of the proteins of interest was carried out using the ECL chemiluminescence detection reagent (GE Healthcare, UK).

To quantify the density of the bands, the software ImageJ (National Institute of Health, NIH, USA) was used, and the results calculated using the relationship between the density of the proteins of interest corrected by the intensity of the protein used as a control (β-actin: 1: 1000, Cell Signaling).

### Statistical analysis

Data were displayed using descriptive measures of position and variability and subjected to analysis of variance (ANOVA) (one-way or two-way) for independent samples when appropriate. When significant differences were found (p < 0.05), a Tukey *post hoc* test was carried out. The level of significance was 5%.

## Results

### Redistribution of experimental groups

The redefinition and characterization of experimental groups in the week 3 showed that 19 animals from the SD group and 18 animals from the HFD group, which had intermediate BW, were excluded. Thus, all analyzes were conducted with four distinct groups: C (n = 15), FC (n = 16), OP (n = 16) and OR (n = 16).

**Fig 2:**
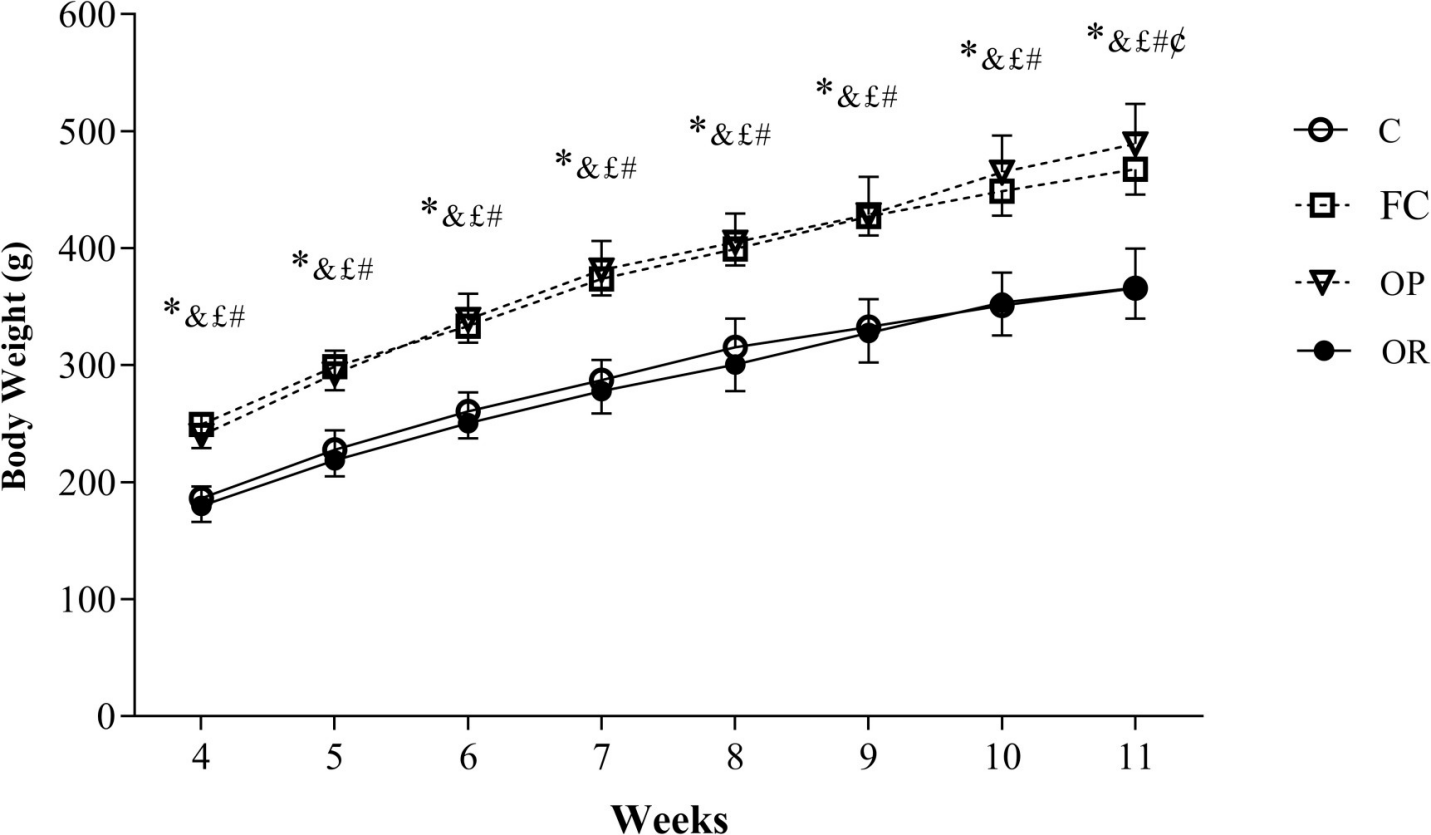
Evolution of body weight after application of the tercile criterion (week 3) for redistribution of groups in Control (C, n = 15), false-control (FC, n = 16); obesity-prone (OP, n = 16) and obesity-resistant (OR, n = 16). Data expressed as mean ± standard deviation. p <0.05 - * OP *vs*. C; ^&^ C *vs*. FC; ^#^ OP *vs*. OR; ^¢^ OP *vs*. FC; ^£^ FC *vs*. OR, repeated measures two-way ANOVA followed by Tukey’s *post-hoc* test.

Fig 2 illustrates the evolution of the animals’ body weight after applying the tercile criterion for group composition, which was performed in the 3^rd^ week of treatment. The results show that BW of OP and FC rats were significantly higher (OP vs. C p < 0.001; OP vs. OR p < 0.001; FC vs. C p < 0.001) when compared to C and OR (4^th^ to 11^th^ week). In addition, FC animals had lower BW in relation to OP only in the 11^th^ week of experimental protocol. There was no significant difference between C and OR during the 8 weeks.

### Characterization of resistance to obesity

Table 1 shows the characterization of OR at the end of the experimental protocol. The results show that the OR animals presented initial (p < 0.001) and final BW (p < 0.001), as well as BW gain smaller than OP and FC groups, however, similar to the C group. When comparing the BW gain in relation to the OP group, the OR, C and FC groups showed a reduction of 25%, 28% and 12.5%, respectively. Regarding body adiposity, it is observed that epididymal (OR vs. C p < 0.0029; OP vs. OR p < 0.001) and retroperitoneal (OR vs. C p < 0.0036; OP vs. OR p < 0.001) fat pads were elevated in OR when compared to group C, but statistically lower compared to OP. There was no statistical difference for these parameters between the OR and FC groups.

**Table 1:** Characterization of resistance to obesity.

Variables	C	FC	OP	OR
**Initial Body Weight(g)**	187 ± 10	250 ± 20*	240 ± 19*	180 ± 14^£^^#^
**Final Body Weight (g)**	366 ± 34	467 ± 22*	489 ± 34*	366 ± 27^£^^#^
**Body Weight Gain (g)**	179 ± 34	217 ± 25*	249 ± 29*^¢^	186 ± 26^£^^#^
**Epididymal Fat (g)**	4.3 ± 1.4	5.5 ± 1.6	10.7 ± 1.3*^¢^	6,1 ± 1.5*^#^
**Retroperitoneal fat (g)**	5.6 ± 2.42	6.9 ± 2.0	16.1 ± 2.9*^¢^	8.7 ± 1.8*^#^
**Visceral fat (g)**	2.7 ± 1,0	3.65 ± 0.9	8.2 ± 2.7*^¢^	3.4 ± 0.8^#^
**Body fat (g)**	12.6 ± 4.4	15.9 ± 4.1	35.0 ± 5.7*^¢^	18.2 ± 3.4*^#^
**Adiposity index (%)**	3.4 ± 1.1	3.4 ± 0.8	7.2 ± 1.0*^¢^	5.0 ± 1.0*^£^^#^
**Food consumption (g/day)**	27.7 ± 1.9	34.8 ± 1.7*	21.1 ± 1.7*^¢^	17.0 ± 1.4*^£^^#^
**Caloric intake (Kcal/day)**	98.8 ± 6.7	124 ± 6.1*	97.4 ± 7.8^¢^	78.0 ± 6.7*^£^^#^
**Feed efficiency (%)**	3.2 ± 0.5	3.1 ± 0.4	4.5 ± 0.6*^¢^	4.2 ± 0.7*^£^

C: Control (n = 15); FC: False-control (n = 16); OR: Obesity-resistant (n = 16) and OP: Obesity-prone (n = 16). Initial body weight at week 4 and final body weight at week 11, respectively. Food consumption, caloric intake and feed efficiency is for the period from week 4 to 11. Data expressed as mean ± standard deviation. p<0.05 –

* vs. C

^#^OP vs. OR

^¢^OP vs. FC

^£^FC vs. OR. One way ANOVA for independent samples, complemented with Tukey’s post-hoc test.

Visceral fat pad did not present a significant difference between the OR, C and FC groups, however, in the OP group it was higher compared to the other groups (p < 0.001). Body fat was increased in the OR group only when compared to the C groups (p < 0.001), but it was similar to FC group. The OP group presented the highest values in this parameter, differing significantly from the other groups (OP vs C p < 0.001; OP vs. OR p < 0.0107; OP vs. FC p < 0.001); AI was elevated in OR group when compared to the C and FC groups (OR vs. C p < 0.003; OR vs FC p < 0.001), however, lower than OP (p < 0.001), which presented high values when compared to all groups (p < 0.001).

Regarding the food profile (4^th^ to 11^th^ week), our results showed that OR and OP rats had lower values of daily food consumption in relation to C (p < 0.001), respectively; on the other hand, the FC group had higher values when compared to OR and OP groups (p < 0.001). It is noteworthy that OR also showed a reduction in daily food consumption when compared to OP (p < 0.001). In addition, OR group had lower daily caloric intake than the other groups (p < 0.001), while the FC group had a higher value for this parameter (p < 0.001); OP group did not present significant difference between the C group. The feeding efficiency of the OR and OP groups did not present statistical difference between them, but OR were increased in relation to the C and FC groups (p < 0.001). There was no statistical difference for feed efficiency between groups C and FC (Table 1).

**Fig 3:**
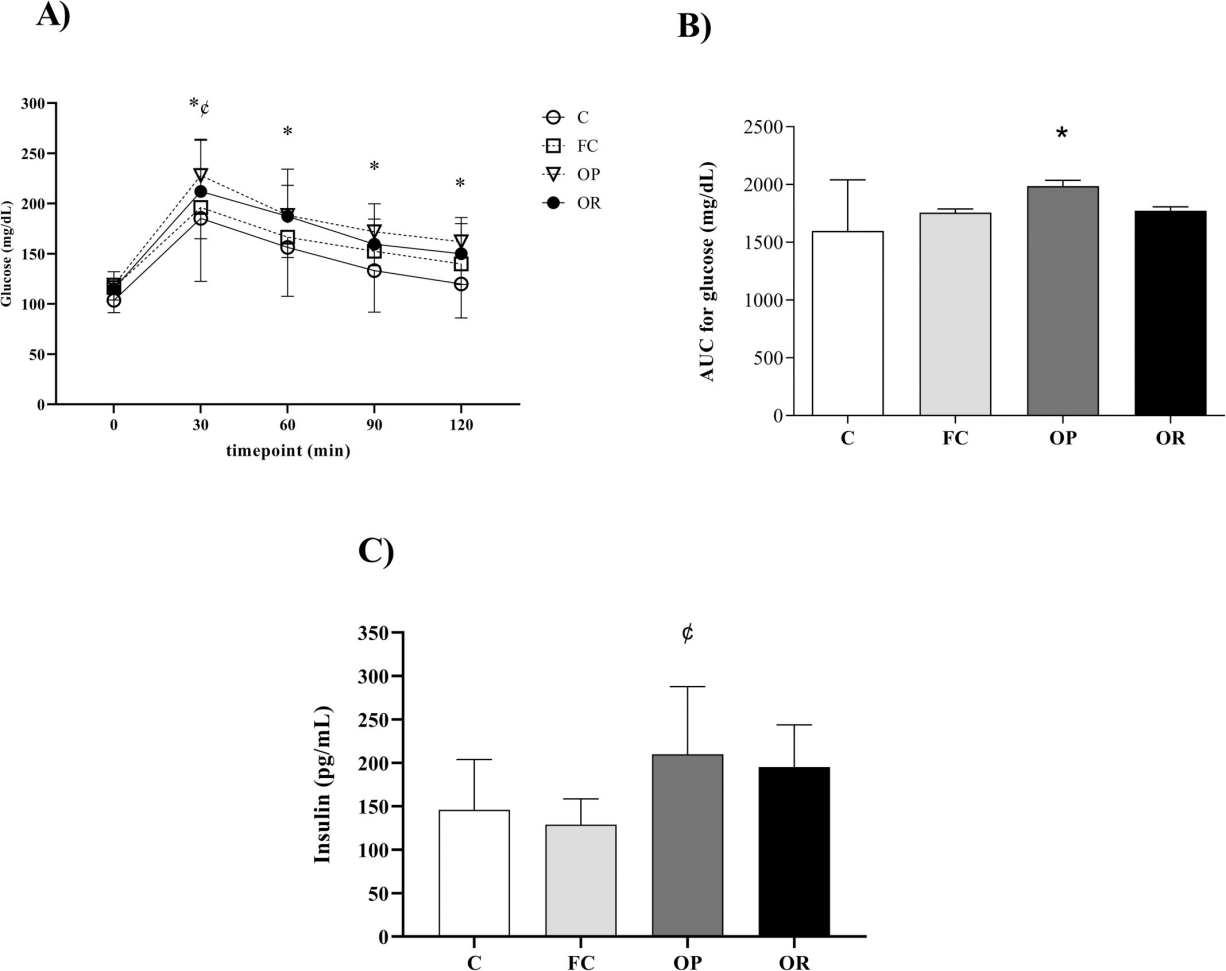
Assessment of glucose metabolism in Control (C, n = 8), false-control (FC, n = 8); obese-prone (OP, n = 8) and obese-resistant (OR, n = 8) rats. A) Blood glucose levels determined during intraperitoneal glucose tolerance testing at week 10; B) Area under the curve (AUC) for glucose and C) Serum insulin concentrations performed after the end of the experimental protocol (week 12). Data are presented as mean ± SD. p<0.05 - * OP vs. C; ^¢^ OP vs FC. Repeated measures two-way ANOVA, followed by Tukey’s *post-hoc* test.

Fig 3 illustrates the glycemic profile at baseline and at 30, 60, 90 and 120 minutes after glucose overload. The results show that after 30 minutes of glucose overload, here was elevated levels of glucose in OP than C rats (p < 0.0028), remaining this difference during the other timepoints (Fig 3A). In addition, there was a significant difference between the OP vs. FC only at the 30-minute timepoint (OP > FC, p < 0.0396), however, this change was not maintained at the other timepoints. In the analysis of AUC for glucose, it can be seen that obesity promoted higher values when compared to group C (p < 0.0007), representing an increase of 24.3% (Fig 3B). It is noteworthy that the condition of OR did not change the glycemic profile, since the OR rats had glycemic and AUC values similar to the other experimental groups. Considering the insulin analysis, the OP group presented higher values only when compared to the FC group (p < 0.350). No differences in these parameters were observed between the other groups (Fig 3C).

**Fig 4:**
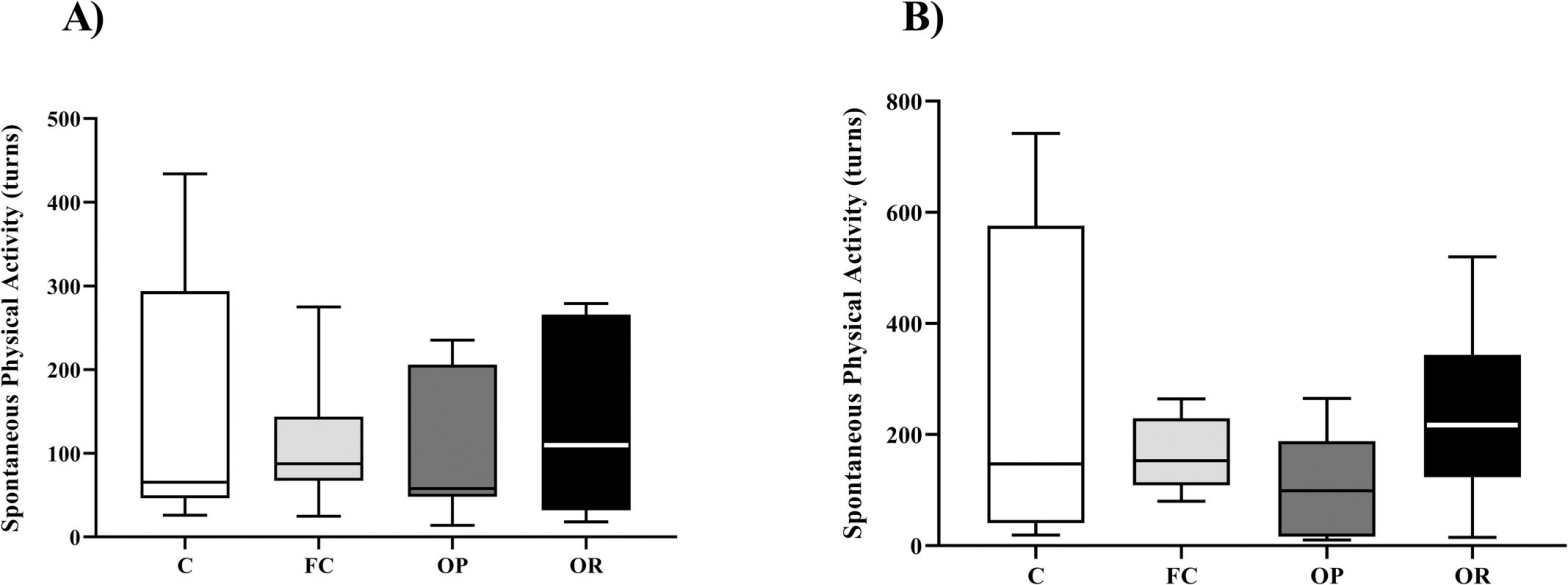
Voluntary physical activity counts measured in weeks 4 and 8. **A)** Turns performed at time 1 for 7 consecutive hours, Control (C, n = 6), false-control (FC, n = 8); obese-prone (OP, n = 7) and obese-resistant (OR, n = 8); **B)** Rotations performed at the moment and for 4 consecutive hours, Control (C, n = 8), false control (FC, n = 6); obesity-prone (OP, n = 8) and obesity-resistant (OR, n = 8). Data are expressed in median and quartiles. One-way non-parametric ANOVA (Kruskal-Wallis) complemented with Dunn’s *post-hoc* test.

Fig 4 illustrates the results obtained in the Voluntary Physical Activity tests, obtained in two moments. Fig 4A showed that groups C and OP have lower median values for voluntary physical activity, however, without statistical difference in week 4 for 7 consecutive hours. On the other hand, the OR group had a higher value than the other groups with no statistical difference. There was no statistical difference in the second moment between the groups for voluntary physical activity counts (Fig 4B).

The results show that the OR rats presented higher HDL levels (22% and 20%) when compared to the OP and FC groups (p < 0.0038; p < 0.0017), respectively, however, similar to C. In addition, OR rats show a reduction of ALT values in relation to C (p < 0.0427), but without statistical difference when compared to the other groups. The AST values were elevated in OR group when compared to the C (p < 0.0297) and FC (p < 0.0406) groups, respectively. OP group presented higher leptin levels when compared to the C (p < 0.0338) and FC (p < 0.0450) groups, respectively. In addition, the results show that there was no significant difference between groups C, FC and OR for this parameter (Table 2). Total cholesterol, total protein, triglycerides and adiponectin were similar between the experimental groups.

**Table 2:** Biochemical and hormonal profile.

Variables	C	FC	OP	OR
**Cholesterol (mg/dL)**	65.4 ± 9.9	62.0 ± 9.8	58.4 ± 12.4	67.6 ± 12.7
**HDL (mg/dL)**	23.3 ± 3.0	22.1 ± 2.2	21.8 ± 3.1	26.6 ± 4.6^£^^#^
**Total proteins (mg/dL)**	5.79 ± 0.29	5.61 ± 0.10	5.73 ± 0.47	5.80 ± 0.25
**Triglycerides (mg/dL)**	32.5 ± 7.7	39.8 ± 16.0	40.1 ± 16.3	36.3 ± 11.1
**AST (mg/dL)**	97.7 ± 24.2	101 ± 31	142 ± 50	146 ± 59*^£^
**ALT (mg/dL)**	54.2 ± 12.0	49.8 ± 5.6	44.9 ± 10.0	44.1 ± 10.1*
**Adiponectin (ng/mL)** ^**¥**^	1.77 ± 0.24	1.75 ± 0.17	1.95 ± 0.31	1.91 ± 0.18
**Leptin (ng/mL)** ^**¥**^	5.62 ± 1.80	6.05 ± 2.47	14.7 ± 8.73*^¢^	8.46 ± 1.61

C: Control (n = 13); FC: False-control (n = 14); OR: Obese-resistant (n = 14); OP: Obese-prone (n = 14). Adiponectin analysis: C (n = 9); FC (n = 9); OR (n = 9); OP (n = 9) and Leptin analysis–

^**¥**^ C (n = 5); FC (n = 5); OR (n = 5); OP (n = 5). HDL: High-intensity lipoprotein; AST: Aspartate aminotransferase; ALT: Alanine aminotransferase. Data expressed as mean ± standard deviation. p<0.05 –

* *vs*. C

^#^OP *vs*. OR

^¢^OP *vs*. FC

^£^FC *vs*. OR. One-way ANOVA for independent samples complemented by Tukey’s *post-hoc* test.

**Fig 5:**
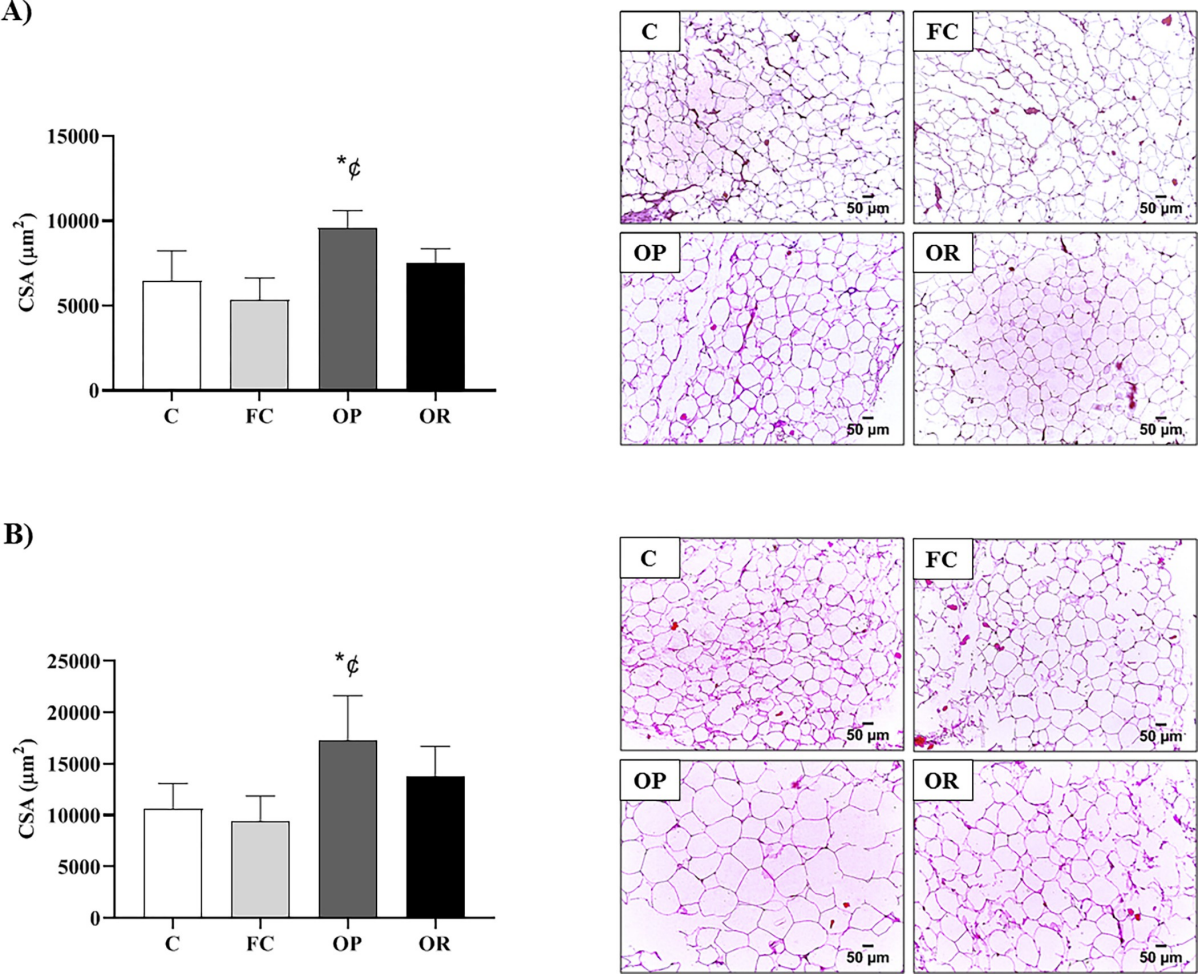
Morphometric analysis of adipose tissues after 11 weeks of experimental protocol. CSA: Cross sectional area. **A)** Hematoxylin-eosin-stained sections of visceral fat; **B)** Hematoxylin-eosin-stained sections of retroperitoneal fat; Control (C, n = 5), false-control (FC, n = 5); obese-prone (OP, n = 5) obese-resistant (OR, n = 5). p<0.05 - *vs. C; ^¢^OP *vs*. FC. Data expressed as mean ± standard deviation. One-way ANOVA for independent samples followed by Tukey’s *post-hoc* test.

Fig 5 shows the results of the morphometric analysis of adipose fat pads. The visceral fat analyzes show that the OP group (9595 ± 1011 μm^2^) had higher CSA areas of adipocytes in relation to C (6466 ± 1781 μm^2^; p < 0.0072) and FC (5311 ± 1317 μm^2^; p < 0.0004), respectively (Fig 5A). Similarly, the analysis of retroperitoneal fat (Fig 5B) shows that the OP group (17253 ± 4390 μm2) presented an increased CSA adipocytes than C (10619 ± 2445 μm^2^; p < 0.0198) and FC (9445 ± 2422 μm^2^; p < 0.0060), respectively. The OR group, in turn, did not show statistical differences for visceral and in relation to all groups.

**Fig 6:**
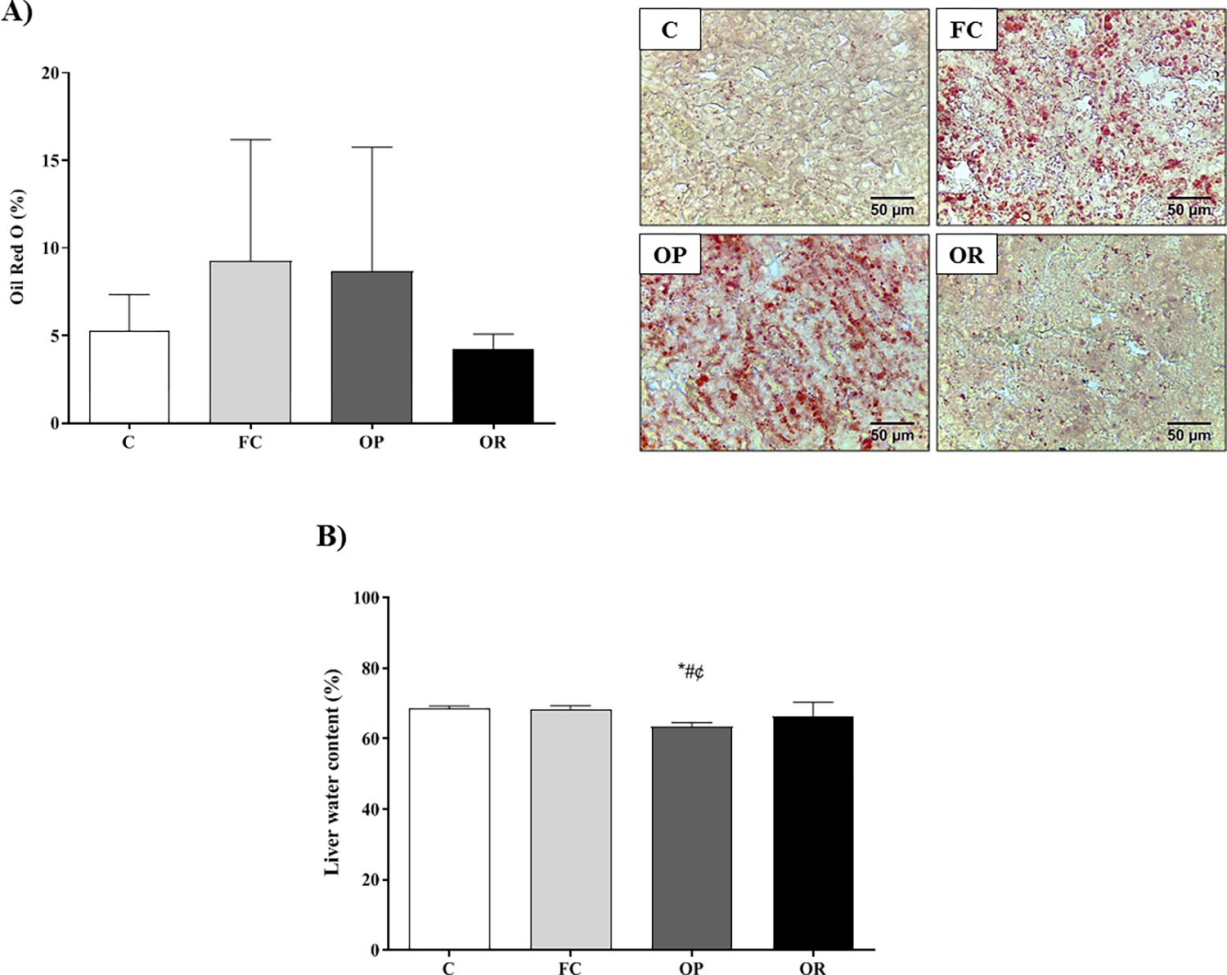
Morphometric analysis and water content in hepatic tissue livers after 11 weeks of experimental protocol. **A)** Fat deposition using the Oil-Red O staining technique taken at 40x magnification; **B)** Water content. Control (C, n = 6), false-control (FC, n = 6); obese-prone (OP, n = 5) and obese-resistant (OR, n = 5). Data expressed as mean ± standard deviation. *p<0.05—OP *vs*. C; ^#^ OP *vs*. OR; ^¢^ OP *vs*. FC. One-way ANOVA for independent samples followed by Tukey’s *post-hoc* test.

Fig 6 illustrates the results of fat deposition and water content in liver tissue by means of Oil-Red-O pigmentation. The results show that there was no significant difference between groups for lipid accumulation in the liver (Fig 6A). However, the OP group had a lower percentage of water in the liver tissue fragments when compared to the C, FC and OR groups (p < 0.05), respectively (Fig 6B).

**Fig 7:**
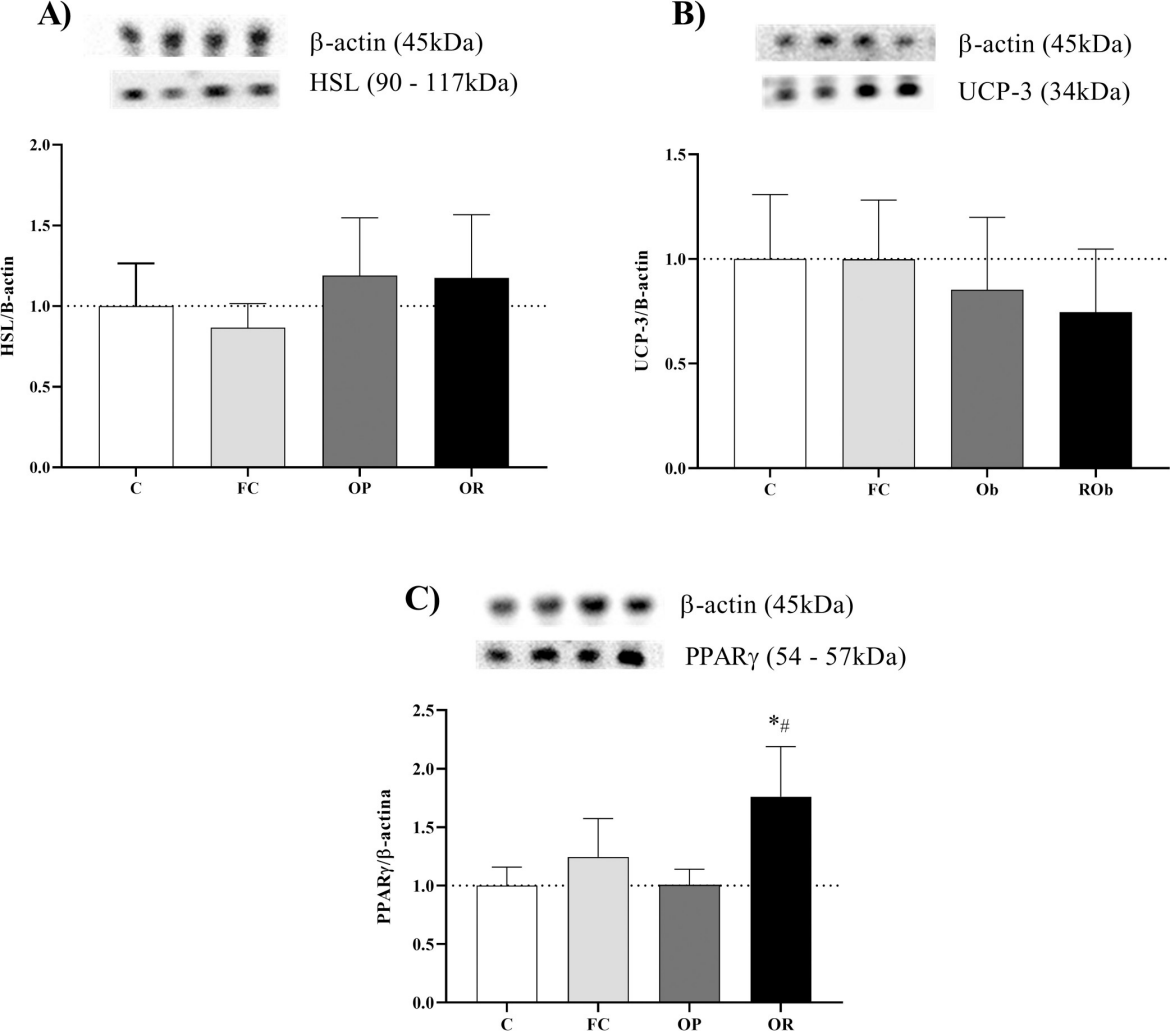
Quantification of soleus skeletal muscle and visceral fat proteins after 11 weeks of experimental protocol. **A)** HSL: Hormone sensitive lipase in VF; **B)** UCP-3: Uncoupling protein 3 (soleus); **C)** PPARℽ: Gamma-type peroxisome proliferator-activated receptor (soleus). Control (C, n = 6), false-control (FC, n = 6); obese-prone (OP, n = 5) and obese-resistant (OR, n = 5). Data expressed as mean ± SD. p<0.05 - *vs. C; ^#^ OP *vs*. OR). One-way ANOVA followed by Tukey’s *post-hoc* test.

The results obtained show that the protein expression of hormone sensitive lipase (HSL) and uncoupling protein 3 (UCP-3) in visceral fat and soleus skeletal muscle, respectively, were not statistically different between groups (Fig 7A and 7B). However, PPARγ protein levels in the soleus skeletal muscle were higher in OR compared to groups C (p < 0.0037) and OP (p < 0.0040), with no significant difference in relation to FC group (Fig 7C).

## Discussion

### Characterization of resistance to obesity

The aim of present study was to characterize metabolically and morphologically the condition of OR, as well as to investigate the probable pathways responsible for lipids oxidation. In addition, it was to investigate if OR rats show elevates spontaneous physical activity. The main findings were that OR promoted ability to resist from developing the Obesity without alterations in glycemic and lipid profiles but was not able to increase the spontaneous physical activity. Furthermore, this ability to resist to Obesity was not due to greater capacity for lipid oxidation as a form of energy production.

The detailing of the composition of the diets is essential to control the conditions of development of obesity and OR [3,20]. The fat profile found in HFD is a relevant variable, as it directly affects body fat gain and the development of metabolic disorders [21]. In the 3^rd^ week of the experimental protocol, the animals exposed to the high-fat diet (HFD) showed a statistical difference in terms of BW when compared to the SD. Therefore, in the current study, it was characterized as the onset of obesity and identification of OR in the 4^th^ week. Woods et al. [22] showed a 10% increase in body mass and a 35–40% gain in body fat in *Long-Evan* rats compared to C animals after 10 weeks. The characterization of OR was evidenced by the absence of changes in BW and weight gain, since the OR animals showed similar results to group C. These results are in according to Jackman, Maclean & Bessen [9], which also did not show any significant difference in the final BW of the OR groups when compared to the OP groups after submission to HFD. The authors suggest the occurrence of different states of energy balance between OR and OP groups, indicating that OP rats had positive energy balance for much of the period with HFD [9]. In this context, both OR and OP animals present different phases of energy expenditure and storage over time, submitted to the consumption of high-fat diets. This shows that their metabolisms respond differently to excess caloric consumption, going through transitions in the priorities of carbohydrate and lipid oxidation, causing different phenotypes of body mass gain and adiposity at different times.

Unlike the findings of the present study, in which the OR animals had lower caloric intake then OP group, we believe that, to optimal characterization of the OR, the animals should have similar caloric intake to OP, not only a lower body mass gain and adiposity (Table 1). In this sense, Akieda-Asai et al. [4] showed that caloric intake was similar between OR and OP rats, but the OR animals developed less body mass gain, that is, the OP group showed greater feed efficiency. Authors suggest that OR has mechanisms that promote greater lipid oxidation [4]. However, these mechanisms are not yet elucidated, and some researchers have demonstrated the involvement of the central nervous system in the phenotype of these [23]. Nevertheless, OR is directly associated with the control of calorie intake and these physiological adaptations seem to be linked to the attempt to maintain energy balance from regulation of the central nervous system, which can be observed within a few days after the introduction of HFD [9]. Corroborating this theory, the leptin results were increased in group OP, but without significant changes in this parameter in OR compared to group C.

Analyzing body adiposity, the OR promoted two distinct behaviors, presenting elevated epididymal and retroperitoneal fat pads than C, but statistically lower in relation to OP rats. It is worth noting that visceral fat, in turn, did not present a significant difference between the OR and C groups, even though both were smaller compared to the OP group. Regarding the morphological analysis, our results have demonstrated that OR did not also promote adipocyte hypertrophy as adaptive response to nutrient excess, being this increase was observed only in the OP group, which indicates that the accumulation of visceral fat is one of the risk factors for mortality. Poret et al. [3] found that OP rats had elevated CSA form adipocytes and greater visceral fat deposits compared to OR. However, HFD intake, *per se*, promoted an increased in expression of pro-inflammatory cytokines in OR, even without the excessive accumulation of body fat. These data support the hypothesis that OP rats are at increased risk of developing comorbidities and that these risks are exacerbated by consuming HFD.

In addition to the incredible ability to resist the development of obesity, the OR model may have cardioprotective characteristics and protection against metabolic disorders, for example, lipotoxicity [1]. These characteristics were also observed in the current study, in which OR rats showed maintenance of glycemic control, normal insulin and improvement in the lipid profile evidenced by normal glycemic values during the glucose tolerance test and higher HDL values, indicating a probable cardioprotection. The levels of total cholesterol, total proteins and triglycerides show that OR does not promote cardiometabolic damage.

Oliveira-Junior et al. [20], when investigating the action of different formulations of high-fat diets, with variations in mono and polyunsaturated fats, also identified an increase in the AUC for glucose only in OP animals, but not in OR and C. It is noteworthy that the condition of OR did not cause damage to the glycemic profile, however, corroborating these findings, Akieda-Asai et al. [4] also visualized lower visceral fat deposition in OR rats compared to OP. These findings suggest that OR rats do not store lipids in excess adipocytes but eliminate them into the circulation. It is noteworthy that the condition of OR did not damage the glycemic profile, however, further study of the aspects of glucose metabolism and the organs involved/affected is essential for advancing the characterization and understanding of this phenomenon.

Another morbidity that has generally been observed in obesity is hepatic steatosis, which is characterized by the accumulation of fat in the liver tissue [24]. The results show that OR did not promote this metabolic disorder. Even though the diet used is efficient to generate elevation of adiposity, it seems not to be aggressive enough to promote hepatic steatosis. Corroborating this argument, Omagari et al. [25] in an attempt to promote hepatic steatosis in *Wistar/ST* rats, using two formulations of high-fat diets with added cholesterol, 1.25% HFC (57.15% - 1.25% cholesterol) and 2.5% HFC (56.18% - 2.5% cholesterol) during 9 weeks of treatment observed non-alcoholic fatty liver disease (steatohepatitis) in both groups, which is more severe than hepatic steatosis. It is interesting to note that both in the study by Omagari et al. [25] and and Huck, Beggs & Apte [26], in which damage to the liver tissue developed, this condition was not caused or preceded by changes in the levels of total cholesterol and circulating triglycerides, as in the present study. However, OR rats showed an increase in circulating AST than C rats, but ALT was reduced. Possibly there may have been an episode of negative feedback in compensation for the increase in AST, since no increases in fat deposition were identified in the liver (Fig 6A). After a liver injury, these enzymes leak into the bloodstream, and thus are considered markers of hepatotoxicity [27]. Nam et al. [28], using C57BL/6J mice subjected to HFD (60% fat) for 6 weeks, have investigated the liver damage caused by HFD in animals that developed Obesity and OR. The authors observed that the OP group had significantly higher values of the two markers when compared to both the C and OR groups, respectively. However, OR, in turn, also presented increased AST and ALT values ​​when compared to group C, showing once again the intermediate characteristic of OR [28]. In the present study, only AST was shown to be increased in OR rats, unlike the study by Nam et al. [28].

Drawing a parallel between the findings by Nam et al. [28] and Jackman, MacLean & Benssen [9], we can infer that at the time of analysis our animals could be in a transitional phase both metabolic and changes in the TG species profile in tissue, so that together, these two factors pegged to 37% fat on HFD, which is not as aggressive as the 60% HD used by Nam et al. [31], may have generated these AST and ALT values reported here. Therefore, it is still unclear whether this was an isolated result or whether OR animals may present some damage to the liver tissue even without the characteristics of steatosis.

Regarding the hormone profile, the current study did not identify that OR condition promotes an elevation in adiponectin levels. However, Barnea, Madar & Froy [29], investigating the expression of genes involved in the adiponectin signaling pathway in adipose tissue and skeletal muscle (target tissue of adiponectin) in mice fed to HFD, observed changes that cause damage to the signaling and expression of these receptors, which, in turn, can lead to “resistance to adiponectin”, as happens in insulin resistance [29].

Leptin is known as the satiety hormone and high circulating amounts are indicative of resistance to its action due to both lower sensitivity and genetic defect in its receptors [30], which leads to hyperphagia and consequently to obesity [31]. Corroborating the theory that obesity-resistant individuals have greater control of caloric intake regulated by the central nervous system, in addition to greater metabolic capacity, which in turn, protect these animals against excessive adiposity and metabolic damage. Our leptin results were increased in the Obesity, but without significant changes in this parameter in OR. Study groups have worked with the hypothesis that OR is due to a complex interaction of several metabolic factors that are activated to a greater or lesser extent in response to environmental factors, for example, contact and maintenance of a high-fat diet [9,32,33]. In this sense, researchers have invested efforts in mapping biomarkers of energy production, storage and consumption systems [2,4,9,24,29,34].

The initial hypothesis raised in the present study was that, in addition to lower body fat deposition, adipocytes from OR animals would also present greater lipolytic efficiency of triglycerides stored in adipose tissue, enhancing the speed of availability of circulating fatty acids, which, in turn, could improve physical performance. The key to this process would be linked to greater amounts of the hormone-sensitive lipase (HSL) enzyme. The regulation of lipolysis is a complex process that involves multiple mechanisms, including lipolytic (β-adrenergic agonists, pirilipins-1, etc.) and their cognate receptors and signaling pathways, particularly involving cyclic AMP and PKA [35,36]. Nevertheless, our findings evidenced the absence of alterations in the protein expression of HSL in adipocytes in OR.

Enzymes that catalyze the esterification of fatty acids into triglycerides are regulated by peroxisome proliferator activated receptors γ (PPARγ) [37]. The PPARγ, as well as the α and β subunits, play a fundamental role in the metabolic systems, among which the gene activation responsible for the transcription of messenger RNAs that signal the beginning of the synthesis of several proteins, among which, the FAT- proteins stand out. The OR promoted an increase in the protein levels of PPARγ in skeletal muscle. The greater activation of PPARγ in adipose tissue seems to promote changes in the lipid profile, so that, despite the gain in body mass, there are improvements in TG levels. Although the present study investigated the content of PPARγ in skeletal muscle, which was increased in the OR group, the results corroborate the researchers’ findings, showing a higher concentration of serum HDL in the OR animals. In the study by Kleiner et al. [38], using transgenic mice knockouts for PPARγ co-activator and gene transcription co-activator 1α (PGC1α), an important co-activator of mitochondrial biogenesis, demonstrate reduced oxidative capacity and insulin resistance [38]. As mentioned above, this seems to be one of the responses for the protective capacity of OR animals against the development of metabolic and cardiometabolic disorders [1].

A possible characteristic of OR would be increased capacity to oxidize fatty acids in skeletal muscles, not only as a mechanism for improving the energy system, but for elimination/cleaning of the body [39]. Thus, the FA would travel to the mitochondria, where they would be oxidized and generating only CO_2_, H_2_O heat (thermogenesis) by the action of UCP-3. Surwit et al. [40] investigated the gene expression of spontaneously Ob (C57BL/6J) and ROb (A/J and C57BL/KsJ) mice submitted to a high-fat diet for two weeks. The authors found no significant differences in the gene expression of messenger RNAs for the synthesis of type 3 uncoupling proteins (UCP-3). Corroborating these findings, in the current study, this hypothesis was not confirmed either, since there was no significant difference in the protein content of UCP-3 in the soleus skeletal muscle.

Another purpose of the current study was to investigate the SPA in the OR condition. Our initial hypothesis was that OR animals would present characteristics favorable to SPA, among which, the improvement of lipid oxidation pathways can be highlighted. This process would have greater influence on both voluntary physical activity and may contribute with greater physical performance.

Researchers have stated that greater spontaneous physical activity, *per se*, is one of the keys that triggers the characteristics of OR [5–7,41]. Some of the main results of the analysis of spontaneous physical activity in these studies were: 1) greater voluntary physical activity verified only by greater vertical displacement [7]; 2) OR animals demonstrated as greater basal spontaneous physical activity, as well as increased SPA-induced orexin A via stimulation of orexin 1 and 2 receptors (OX1R and OX2R) in the hypothalamus [6]. In this sense, orexigenic neurons are important elements for motor activity, pointing out that they play a key role in spontaneous physical activity [5]. This context could explain, in part, the increase in body mass and fat mass gain in HFD interventions, even when caloric intake is similar to C group. Nevertheless, our results showed that OR did not increase the SPA parameters in both moments. Corroborating these findings, Jackman, Maclean & Bessen [9] also did not identify significant differences in SPA. In addition, the authors stated that they oppose the idea that increased spontaneous physical activity plays an important role in protecting against body mass gain in OR rats [9]. In the current study, we choose to test whether OR animals voluntarily seek exercise, the running wheels were attached to the animals’ boxes, giving them the option to go to the wheels and run or not. For this reason, we believe that no significant differences were found.

## Conclusion

Resistance to Obesity has prevented that OR animals from developing Obesity, but was not able to increase the spontaneous physical activity. However, insufficient evidence was found to state that this ability to resist to Obesity is directly related to greater metabolic and oxidative capacity of fatty acids.

## Limitations

As for the greater spontaneous physical activity of OR animals, several details can be modified in future studies, bringing subsidies and more enlightening answers. Some of the proposed changes are: 1) Increase in the number of animals and test moments; 2) Add the quantification of counts performed on the wheels with the movement in the box, as an animal can perform 100 uninterrupted counts or can perform 5 rounds of 20 turns with intervals between each, this represents completely different physical activity characteristics and 3) evaluate the locomotor activity of the animals in the box.

## Supporting information

S1 Table Characterization of resistance to obesity. (XLS)

S2 Table Biochemical and hormonal profile. (XLSX)

S1 Fig Evolution of body weight. (XLS)

S2 Fig Assessment of glucose metabolism. (XLS)

S3 Fig Voluntary physical activity counts measured in weeks 4 and 8. (PDF)

S4 Fig Morphometric analysis of adipose tissues after 11 weeks of experimental protocol. (XLS)

S5 Fig Morphometric analysis and water content in hepatic tissue livers after 11 weeks of experimental protocol. (XLS)

S6 Fig Quantification of soleus skeletal muscle and visceral fat proteins after 11 weeks of experimental protocol. (XLSX)
