# Supplementary material for: Resistance to obesity prevents obesity development without increasing spontaneous physical activity and not directly related to greater metabolic and oxidative capacity
Source: PLoS One. 2022 Aug 11;17(8):e0271592. doi: 10.1371/journal.pone.0271592 (PMC9371322; doi:10.1371/journal.pone.0271592)
Supplement: S3 Fig — (PDF) [file pone.0271592.s005.pdf]

Descriptive statistics		A	B	C	D
		C	FC	OP	OR
1	Number of values	6	8	7	8
2					
3	Minimum	26.00	25.00	14.00	18.00
4	25% Percentile	46.25	67.25	48.00	32.00
5	Median	65.50	87.50	58.00	111.0
6	75% Percentile	293.8	143.8	206.0	265.8
7	Maximum	434.0	275.0	235.0	279.0
8	Range	408.0	250.0	221.0	261.0
9					
10	Mean	148.5	112.4	116.9	139.5
11	Std. Deviation	160.8	76.54	92.88	115.5
12	Std. Error of Mean	65.65	27.06	35.11	40.82
13					
14	Coefficient of variation	108.3%	68.11%	79.49%	82.77%
